# Mutations of Arabidopsis *TBL32* and *TBL33* Affect Xylan Acetylation and Secondary Wall Deposition

**DOI:** 10.1371/journal.pone.0146460

**Published:** 2016-01-08

**Authors:** Youxi Yuan, Quincy Teng, Ruiqin Zhong, Marziyeh Haghighat, Elizabeth A. Richardson, Zheng-Hua Ye

**Affiliations:** 1 Department of Plant Biology, University of Georgia, Athens, GA, 30602, United States of America; 2 Department of Pharmaceutical and Biomedical Sciences, University of Georgia, Athens, GA, 30602, United States of America; Institute of Genetics and Developmental Biology, Chinese Academy of Sciences, CHINA

## Abstract

Xylan is a major acetylated polymer in plant lignocellulosic biomass and it can be mono- and di-acetylated at *O*-2 and *O*-3 as well as mono-acetylated at *O*-3 of xylosyl residues that is substituted with glucuronic acid (GlcA) at *O*-2. Based on the finding that ESK1, an *Arabidopsis thaliana* DUF231 protein, specifically mediates xylan 2-*O*- and 3-*O*-monoacetylation, we previously proposed that different acetyltransferase activities are required for regiospecific acetyl substitutions of xylan. Here, we demonstrate the functional roles of TBL32 and TBL33, two ESK1 close homologs, in acetyl substitutions of xylan. Simultaneous mutations of *TBL32* and *TBL33* resulted in a significant reduction in xylan acetyl content and endoxylanase digestion of the mutant xylan released GlcA-substituted xylooligomers without acetyl groups. Structural analysis of xylan revealed that the *tbl32 tbl33* mutant had a nearly complete loss of 3-*O*-acetylated, 2-*O*-GlcA-substituted xylosyl residues. A reduction in 3-*O*-monoacetylated and 2,3-di-*O*-acetylated xylosyl residues was also observed. Simultaneous mutations of *TBL32*, *TBL33* and *ESK1* resulted in a severe reduction in xylan acetyl level down to 15% of that of the wild type, and concomitantly, severely collapsed vessels and stunted plant growth. In particular, the S2 layer of secondary walls in xylem vessels of *tbl33 esk1* and *tbl32 tbl33 esk1* exhibited an altered structure, indicating abnormal assembly of secondary wall polymers. These results demonstrate that TBL32 and TBL33 play an important role in xylan acetylation and normal deposition of secondary walls.

## Introduction

Plant cell walls consist of cellulose, hemicelluloses, pectins, lignin, and proteins, the proportion of which may vary among different species and between different tissues and cell types in the same species. The wall polymers, including hemicelluloses (xyloglucan, xylan and glucomannan), pectins (homogalacturonan, rhamnogalacturonan I and rhamnogalacturonan II), and lignin, are often acetylated [1,2]. Acetylation of wall polymers plays important roles in the mechanical strength of cell walls, cell elongation, and disease resistance as exemplified in several Arabidopsis mutants and overexpression lines. A reduction in xylan acetylation causes a decrease in secondary wall thickening and a deformation of vessels [3,4], deacetylation of pectin by overexpression of a pectin acetylesterase results in a defect in cell elongation [5], and a reduction in the acetylation of xylan and pectin by overexpression of acetylesterases leads to an increased resistance to fungal infection [6,7]. Recently, an increased attention has been paid to the study of acetylation of wall polymers because acetylation contributes to the recalcitrance of lignocellulosic biomass for its conversion into liquid biofuels. Xylan acetylation in lignocellulosic biomass hinders the access and hydrolysis of xylan by xylanlytic enzymes, which negatively impacts the hydrolysis of cellulose into sugars [8]. Furthermore, the acetyl groups released during biomass pretreatments are inhibitory to microorganisms used for sugar fermentation [9]. It is, therefore, imperative to uncover the biochemical mechanisms underlying the acetylation of wall polymers, the knowledge of which may help design strategies to modify lignocellulosic biomass tailored for biofuel production.

The position of acetyl groups and the degree of acetylation vary among different wall polymers. Xyloglucan, the major hemicellulose in dicot primary walls, consists of a linear chain of glucosyl residues that are substituted with xylose, xylose-galactose disaccharide, and xylose-galactose-fucose trisaccharide in a regular pattern. Acetyl substitutions of xyloglucan occur on galactosyl residues; they may be monoacetylated at *O*-3, *O*-4 or *O*-6 and diacetylated at both *O*-3 and *O*-6 or at both *O*-4 and *O*-6. The degree of *O*-acetylation of galactosyl residues. i.e., the molecular ratio of acetyl groups to galactosyl residues, is about 0.8 [10]. An exception is xyloglucan from tomato in which the glucosyl backbone is substituted with xylose, xylose-galactose, and xylose-arabinose disaccharides, and the acetyl moieties are attached to galactosyl residues at *O*-6, arabinosyl residues at *O*-5, and/or glucosyl residues at *O*-6 [11]. Xylan, the major hemicellulose in dicot secondary walls, is composed of a linear chain of xylosyl residues that are branched with glucuronic acids (GlcA), 4-*O*-methylglucuronic acid (MeGlcA), or arabinose depending on plant species. Xylan may be mono- and di-acetylated at *O*-2 and *O*-3 of xylosyl residues, and monoacetylated at *O*-3 of xylosyl residues branched with GlcA/MeGlcA at *O*-2 [12]. The degree of acetyl substitutions in xylan varies from 0.3 to 0.6 depending on plant species [3, 12–18]. Glucomannans from aspen and birch wood are acetylated at *O*-2 or *O*-3 of mannose residues with a degree of acetylation of about 0.3 [19]. In pectins (homogalacturonan and rhamnogalacturonan I), the acetyl moieties are attached to galacuronic acid residues at *O*-2 and/or *O*-3 [20,21]. In addition to polysaccharides, lignin from angiosperms is acetylated at the γ-carbon of the side chain of syringyl and guaiacyl units with a degree of acetylation ranging from 0.06 to 0.8 depending on plant species [22].

Recent genetic and biochemical analyses in Arabidopsis have revealed the involvement of three groups of proteins, RWAs (Reduced Wall Acetylation), DUF231 proteins, and AXY9 (Altered Xyloglucan9), in cell wall polysaccharide acetylation. The Arabidopsis genome harbors four *RWA* genes, which encode proteins homologous to the fungal CAS1 protein involved in acetylation of glucuronoxylomannan, the major capsular polysaccharide [23]. The *RWA* genes are expressed in cells undergoing secondary wall thickening although *RWA2* also exhibits a low level of expression in parenchyma cells. Simultaneous mutations of the four *RWA* genes result in a reduction in xylan acetylation and a reduced mechanical strength of stems [24]. A reduction in acetylation of xyloglucan and glucomannan was also reported in the *rwa* mutants [25]. Although the fungal protein CAS1 contains a putative acetyltransferase domain in addition to multiple transmembrane helices, RWAs only possess multiple transmembrane helices, and thus they were proposed to be putative transporters for acetyl donors [1]. Mutation of *AXY9* has been demonstrated to cause a reduction in acetylation of both xyloglucan and xylan, a defect in plant growth, and a deformation of vessels. It was suggested that AXY9 might be involved in providing the supply of acetyl donor substrates for acetylation of multiple polysaccharides [26]. Three DUF231 proteins, including AXY4/TBL27, AXY4L/TBL22, and ESK1 (ESKIMO1)/TBL29, have been implicated in acetylation of xyloglucan or xylan. There are 46 DUF231 members in Arabidopsis and they are characterized by the presence of two conserved domains, the DUF231 domain and the TBL (Trichome Birefringence-Like) domain [27]. Mutations of *AXY4* and *AXY4L* result in a loss of acetyl groups in xyloglucan from leaves and roots and from seeds, respectively. AXY4 and AXY4L are proposed to be putative acetyltransferases catalyzing *O*-acetylation of xyloglucan [28]. The *esk1* mutant was first identified from a mutant screening for freezing tolerance [29] and the *ESK1* gene was expressed specifically in xylem and fiber cells [3]. The *esk1* mutant exhibits a defect in xylan 2-*O*- and 3-*O*-monoacetylation and a reduction in xylan acetyltransferase activity [3,4], and in vitro assay confirmed that ESK1 is a xylan acetyltransferase catalyzing 2-*O*- and 3-*O*-monoacetylation of xylosyl residues [30].

Based on the finding that *esk1* causes a specific defect in xylan monoacetylation at *O*-2 and *O*-3, we have previously proposed that different acetyltransferase activities might mediate monoacetylation, diacetylation, and monoacetylation of xylosyl residues substituted at *O*-2 with GlcA/MeGlcA and that ESK1 close homologs are candidates for such acetyltransferase activities [3]. Here, we demonstrate roles of TBL32 and TBL33, two ESK1 close homologs, in xylan acetylation. We show that simultaneous mutations of *TBL32* and *TBL33* cause a nearly complete loss of acetyl groups at *O*-3 of xylosyl residues that are also branched with GlcA/MeGlcA at *O*-2, indicating that TBL32 and TBL33 are putative acetyltransferases involved in acetyl substitutions of 2-*O*-GlcA-substituted xylosyl residues. In addition, the *tbl32 tbl33* double mutant exhibits a partial reduction in 3-*O*-monacetylation and 2,3-di-*O*-acetylation of xylosyl residues. Our findings provide further genetic evidence supporting the hypothesis that different acetyltransferase activities might mediate the regiospecific acetyl substitutions of xylosyl residues in xylan.

## Results

### Expression of *TBL33* Is Associated with Cells Undergoing Secondary Wall Thickening

**Fig 1:**
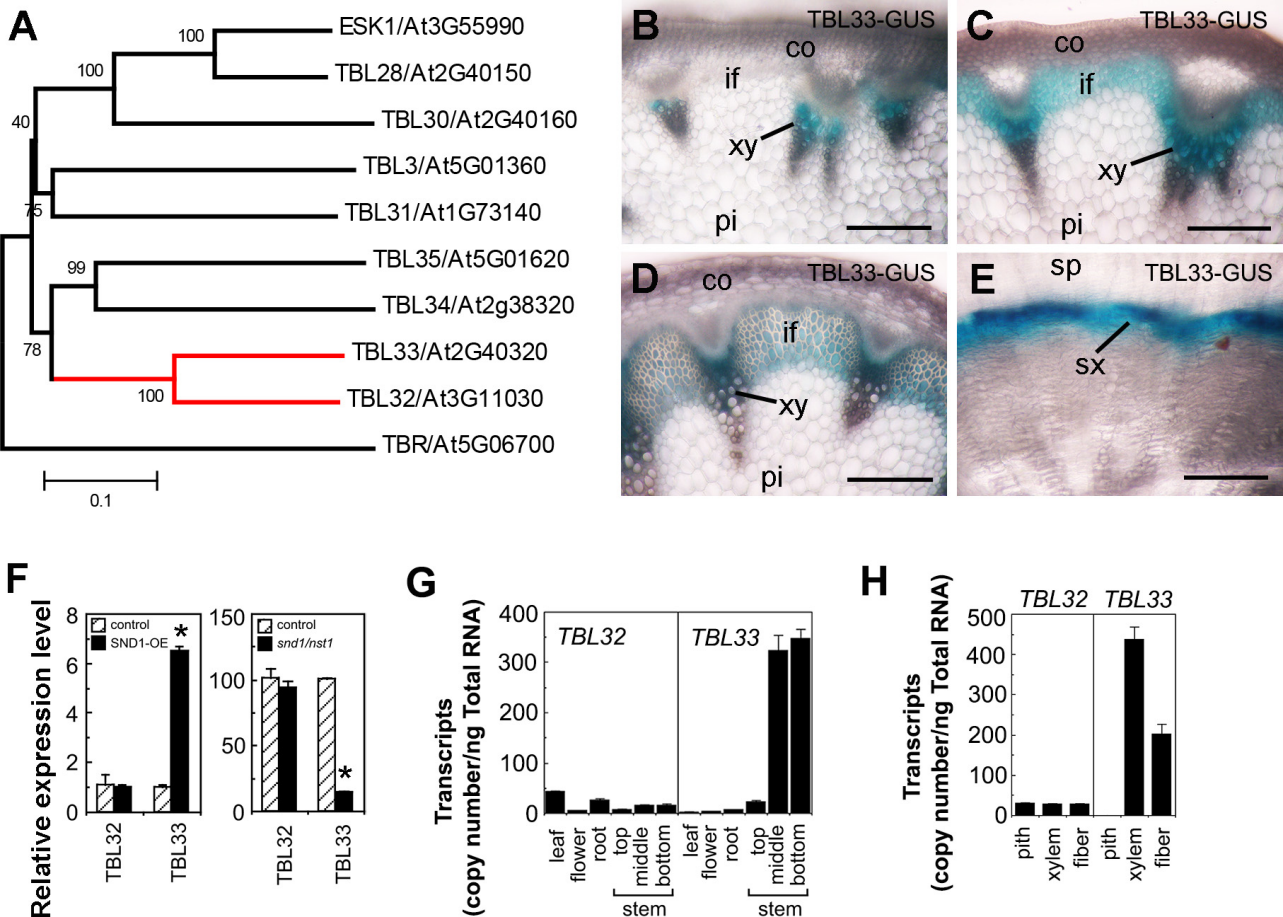
Expression analysis of the Arabidopsis *TBL32* and *TBL33* genes. (A) Phylogenetic relationship of TBL32, TBL33, and other ESK1 homologs. TBR, another member of the Arabidopsis DUF231 family, is also included in the phylogenetic analysis. The phylogenetic tree was constructed with the neighbor-joining algorithm. The 0.1 scale denotes 10% change, and the bootstrap values resulted from 1,000 replicates are presented in percentages at the nodes. (B) to (E) Expression patterns of *TBL33* in Arabidopsis inflorescence stems and root-hypocotyls. Cross sections of the top stem (C), middle stem (D), bottom stem (E), and root-hypocotyl (F) of TBL33::TBL33-GUS plants were stained for GUS activity (blue). co, cortex; if, interfascicular fiber; pi, pith; sp, secondary phloem; sx, secondary xylem; xy, xylem. Bars = 165 μm. (F) Quantitative PCR analysis of the expression of *TBL32* and *TBL33* in the SND1 overexpressors (SND1-OE) (left panel) and the *snd1 nst1* double mutant (right panel). The expression of each gene in the wild type was used as a control by setting to 1 for SND1 overexpressors and by setting to 100 for the *snd1 nst1* double mutant. Asterisks indicate statistically significant differences compared with the control (*p* < 0.001). (G) Quantitative PCR analysis of the expression of *TBL32* and *TBL33* in different Arabidopsis organs. (H) Quantitative PCR analysis of the expression of *TBL32* and *TBL33* in pith, xylem and interfascicular fibers in wild-type stems. The expression level in (G) and (H) was presented as transcript copy numbers per ng total RNA. Error bars denote SD of three biological replicates for each sample.

Among the 46 members in the Arabidopsis DUF231 family, eight of them are close homologs of ESK1 (Fig 1A) [27]. Here, we focused on functional characterization of two of them, TBL32 and TBL33, which are closely grouped together (Fig 1A). To investigate their possible roles in xylan acetylation, we first examined whether their expression was associated with secondary wall biosynthesis. Quantitative PCR analysis revealed that *TBL33* expression was highly upregulated by overexpression of SND1 (Fig 1F), a master transcriptional switch activating the biosynthetic genes of xylan, cellulose, and lignin [31]. Conversely, the expression of *TBL33* was reduced in the *snd1 nst1* stems (Fig 1F), in which secondary walls were absent in fibers [32]. The expression of *TBL32* was not altered in the SND1 overexpressors or the *snd1 nst1* double mutant. Expression analysis in different Arabidopsis organs showed that *TBL33* was preferentially expressed in inflorescence stems undergoing secondary wall deposition, whereas *TBL32* expression level was relatively low and similar in different organs (Fig 1G).

We used the β-glucuronidase (GUS) reporter gene to examine further the expression patterns of *TBL32* and *TBL33* in transgenic Arabidopsis plants. The entire gene sequences of *TBL32* and *TBL33*, including the 5’ upstream sequence, the coding region, and the 3’ downstream sequence, were used for the generation of the *TBL32*::*TBL32-GUS* and *TBL33*::*TBL33-GUS* reporter genes in order to ensure that the expression patterns of the GUS reporter gene genuinely represented those of the endogenous genes. Examination of the stems of *TBL33*:*TBL33-GUS* transgenic plants demonstrated that in early elongating internodes where protoxylem was the only secondary wall–containing cells being produced [33], the GUS staining was only present in the protoxylem (Fig 1B). In internodes that were near the cessation of elongation (Fig 1C) and in nonelongating internodes (Fig 1D), where secondary wall deposition occurred in fibers and xylem, intensive GUS staining for *TBL33* was observed in both interfascicular fibers and developing metaxylem. In root-hypocotyls undergoing secondary growth, a few layers of developing secondary xylem cells displayed heavy GUS staining for *TBL33* (Fig 1E). We were unable to observe GUS staining for *TBL32*, probably due to its low expression level. To find out what cell types *TBL32* was expressed, we used quantitative PCR analysis to investigate its expression in three cell types, including pith cells, xylem cells and interfascicular fibers that were laser-microdissected from non-elongating stem internodes (Fig 1H). The results showed that a low-level expression of *TBL32* was observed in all of three cell types examined, whereas a high-level expression of *TBL33* was detected only in xylem cells and interfascicular fibers but not in pith cells, which was consistent with the GUS reporter gene expression data (Fig 1B to 1E). These expression studies reveal that while a low-level *TBL32* expression is linked with both primary wall- and secodnary wall-forming cells, a high-level *TBL33* expression is specifically associated with secondary wall-forming cells.

### TBL32 and TBL33 Are Localized in the Golgi

**Fig 2:**
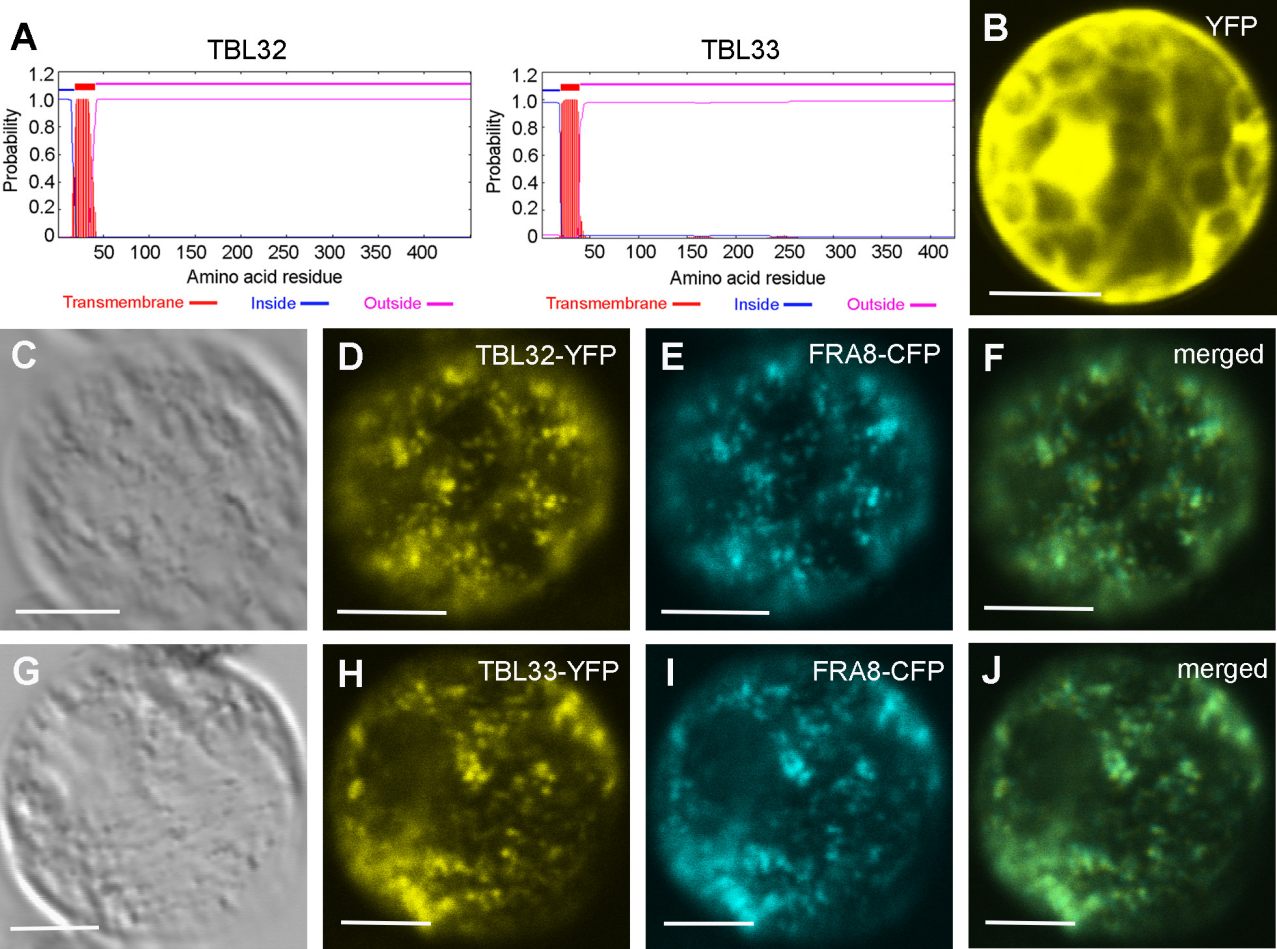
TBL32 and TBL33 are co-localized with FRA8. Arabidopsis leaf protoplasts expressing fluorescent protein-tagged fusion proteins were visualized for fluorescent signals with a laser confocal microscope. (A) TBL32 and TBL33 are type II membrane proteins based on the TMHMM2.0 program. Outside, the noncytoplasmic side of the membrane (Golgi); inside, the cytoplasmic side of the membrane. (B) An Arabidopsis protoplast expressing YFP alone showed the distribution of the signals throughout the cytoplasm. (C) to (F) An Arabidopsis protoplast (C) co-expressing TBL32-YFP (D) and the Golgi-localized FRA8-CFP (E). Note the overlap of the signals of TBL32-YFP and FRA8-CFP (F). (G) to (J) An Arabidopsis protoplast (G) co-expressing TBL33-YFP (H) and FRA8-CFP (I). Note the overlap of the signals of TBL33-YFP and FRA8-CFP (J). Bars in (B) to (J) = 12 μm.

Sequence analysis of TBL32 and TBL33 using the TMHMM2.0 program (http://www.cbs.dtu.dk/service/TMHMM-2.0) indicated that they are membrane proteins with a single transmembrane helix, a short N-terminal tail on the cytoplasmic side, and a large C-terminal domain on the noncytoplasmic side (Fig 2A). To investigate their subcellular localizations, yellow fluorescent protein-tagged TBL32 and TBL33 and cyan fluorescent protein-tagged Golgi marker FRA8 [34] were co-expressed in Arabidopsis protoplasts. Visualization of the fluorescent signals showed that while the protoplasts expressing YFP alone displayed signals throughout the cytoplasm (Fig 2B), those expressing TBL32-YFP and TBL33-YFP exhibited a punctate pattern, which was overlapped with that of FRA8-CFP (Fig 2C–2J). These results demonstrate that TBL32 and TBL33 are targeted to the Golgi.

### Simultaneous Mutations of *TBL32*, *TBL33*, and *ESK1* Result in Stunted Plant Growth, Disorganized Secondary Walls and Severely Collapsed Vessels

**Fig 3:**
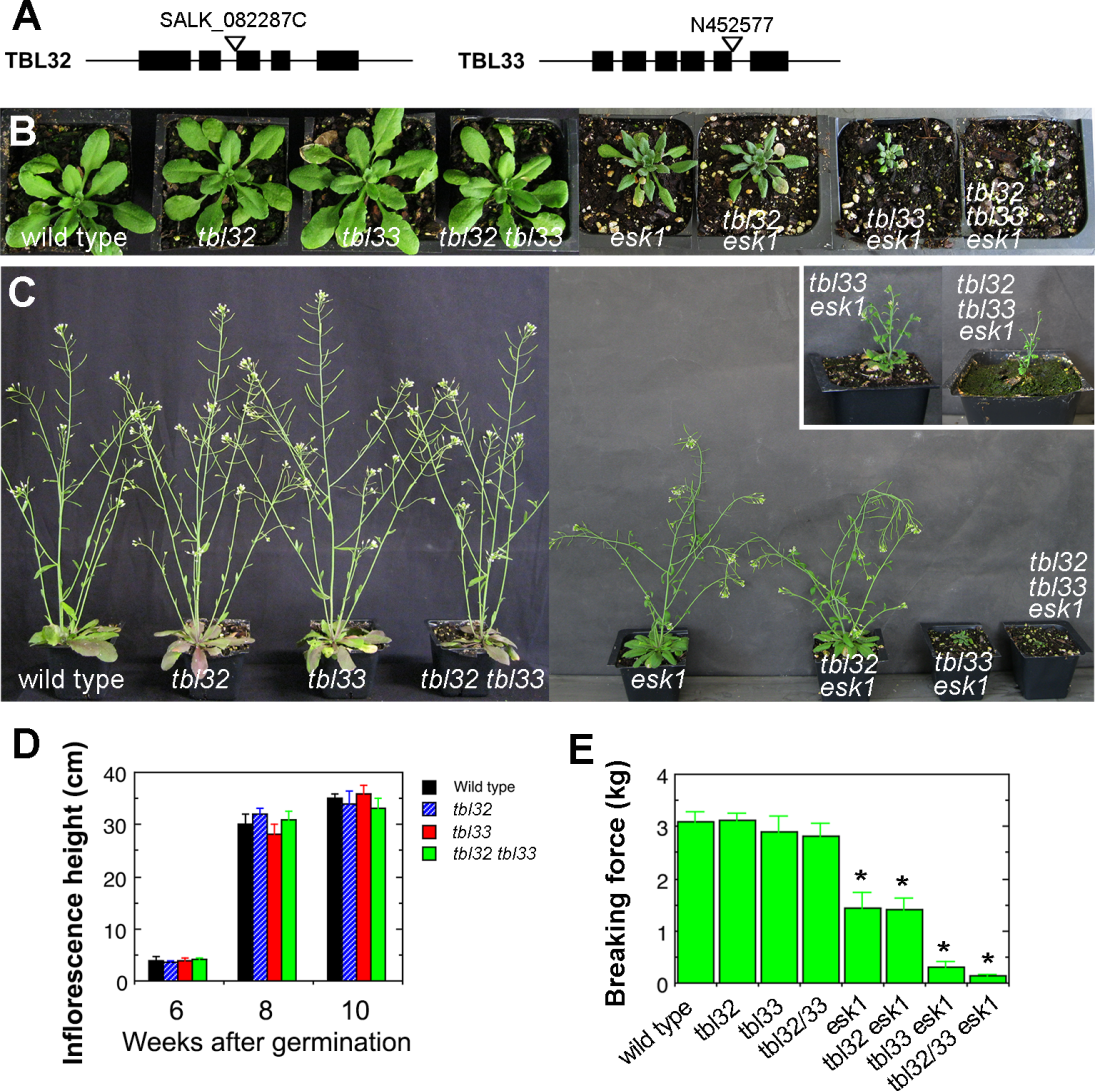
Effects of mutations of *TBL32*, *TBL33*, and *ESK1* on stem mechanical strength and plant growth. (A) The sites of T-DNA insertions in the *TBL32* and *TBL33* genes. Filled boxes represent exons. (B) Morphology of 4-week-old seedlings of the wild type and various mutants. (C) Morphology of 8-week-old wild-type and mutant plants. Inset shows the images of a 12-week-old plant of *tbl33 esk1* and a 16-week-old plant of *tbl32 tbl33 esk1*. Note the extremely dwarfed plants of *tbl33 esk1* and *tbl32 tbl33 esk1*. (D) Measurement of inflorescence stem heights during different developmental stages in the wild type, *tbl32*, *tbl33*, and *tbl32 tbl33* mutants. (E) Stem strength measurement in the wild type and various mutants. Basal parts of mature stems were measured for their breaking force. Error bars in (D) and (E) are SD of measurements from 20 independent plants. Asterisks in (E) indicate statistically significant differences compared with the wild type (*p* < 0.001).

To study the functions of TBL32 and TBL33, we obtained T-DNA insertion mutants of *TBL32* and *TBL33* and generated double and triple mutants of *TBL32*, *TBL33*, and *ESK1*. The T-DNA insertions occurred at the second intron of *TBL32* and at the fifth intron of *TBL33* (Fig 3A). When gene-specific primers (from second and third exons for *TBL32*; from fifth and sixth exons for *TBL33*) were used to PCR-amplify the transcripts of *TBL32* or *TBL33* using total RNA isolated from mutant stems, we did not detect any PCR amplification products after 40 cycles. In contrast, we detected PCR amplification products of *TBL32* or *TBL33* transcripts using total RNA isolated from wild-type stems after 28 cycles. These results indicate that no normal *TBL32* or *TBL33* transcripts were present in the respective mutant lines. Phenotypic analysis showed that unlike the *esk1* mutant, which had a prominent reduction in plant growth [3,4], the single and double mutants of *TBL32* and *TBL33* did not display any apparent alterations in plant growth (Fig 3B, 3C and 3D). Although the plant size and stem strength of the *tbl32 esk1* double mutant are similar to those of *esk1* (Fig 3B, 3C and 3E), the *tbl33 esk1* double mutant exhibited a drastic reduction in plant growth. The rosette sizes of *tbl33 esk1* were about 3 times smaller than those of *esk1* (Fig 3B). In addition, the growth of inflorescence stems was significantly delayed and inhibited in *tbl33 esk1*; only small inflorescences developed after 12 weeks of prolonged growth (Fig 3C, inset) and their stem strength was reduced by 5-fold compared with that of *esk1* (Fig 3E). Furthermore, simultaneous mutations of *TBL32*, *TBL33*, and *ESK1* reulted in a much more severe defect in plant growth and stem strength (Fig 3B, 3C and 3E). After 16 weeks of prolonged growth, the *tbl32 tbl33 esk1* triple mutant plants only produced tiny inflorescences (Fig 3C, inset) and their stem strength was reduced by 11-fold compared with that of *esk1* (Fig 3E).

**Fig 4:**
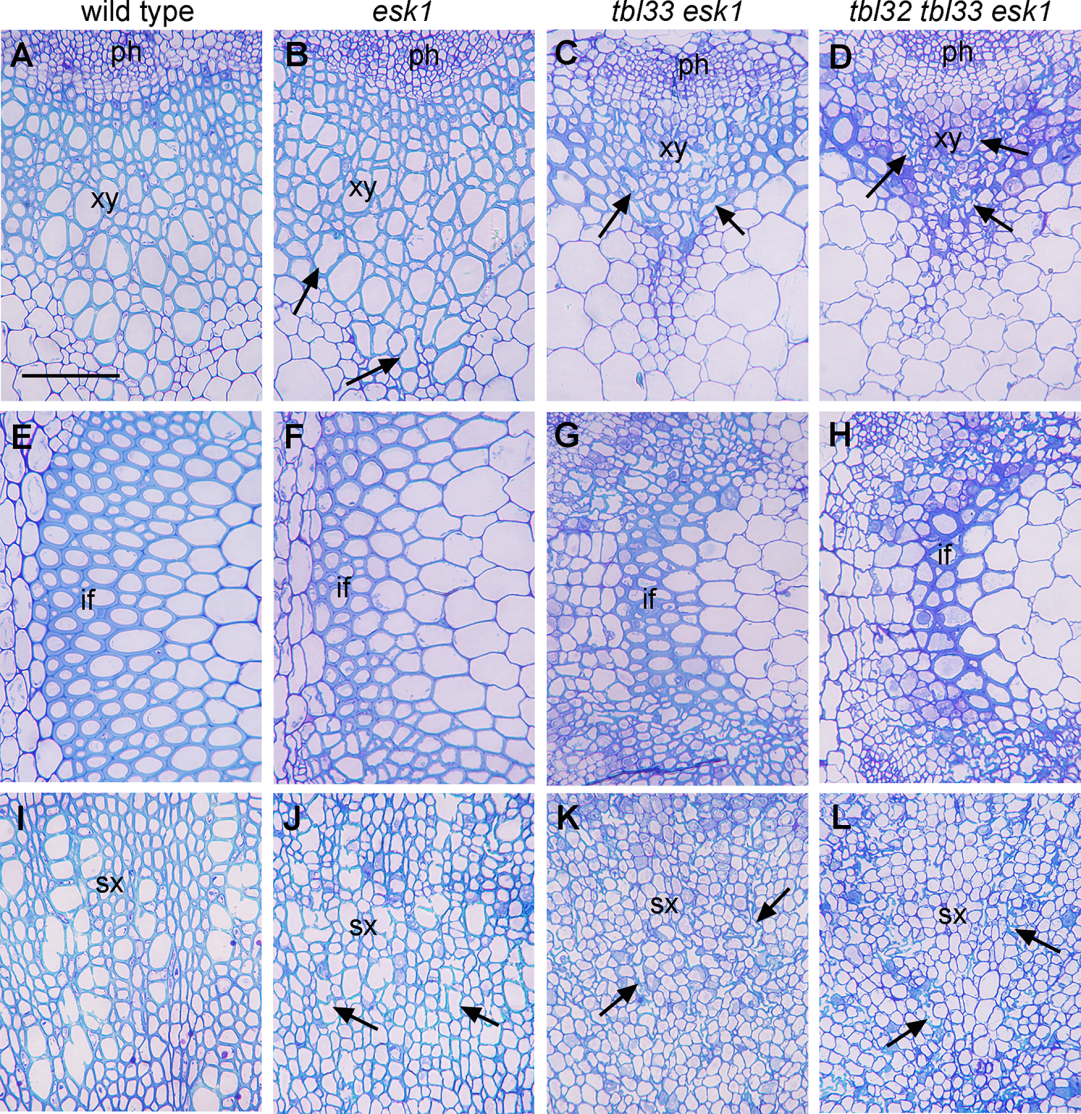
Effects of mutations of *TBL32*, *TBL33*, and *ESK1* on vessel morphology and secondary wall thickening. The bottom internodes of stems and the root-hypocotyls of 8-week-old wild-type and *esk1* plants, 12-week-old *tbl33 esk1* plants, and 16-week-old *tbl32 tbl33 esk1* plants were sectioned for visualization of anatomical structures. (A) to (D) Cross sections of stem xylem bundles showing vessels (arrows) with a mild deformation in *esk1* (B) and a severe deformation in *tbl33 esk1* (C) and *tbl32 tbl33 esk1* (D) compared with the wild type (A). (E) to (H) Cross sections of stem interfascicular regions showing defective secondary wall thickening in *esk1* (F), *tbl33 esk1* (G), and *tbl32 tbl33 esk1* (H) compared with the wild type (E). (I) to (L) Cross sections of root-hypocotyls showing vessels (arrows) with a mild deformation in *esk1* (J) and a severe deformation in *tbl33 esk1* (K) and *tbl32 tbl33 esk1* (L) compared with the wild type (I). if, interfascicular fiber; ph, phloem; sx, secondary xylem; xy, xylem. Bar in (A) = 68 μm for (A) to (L).

**Fig 5:**
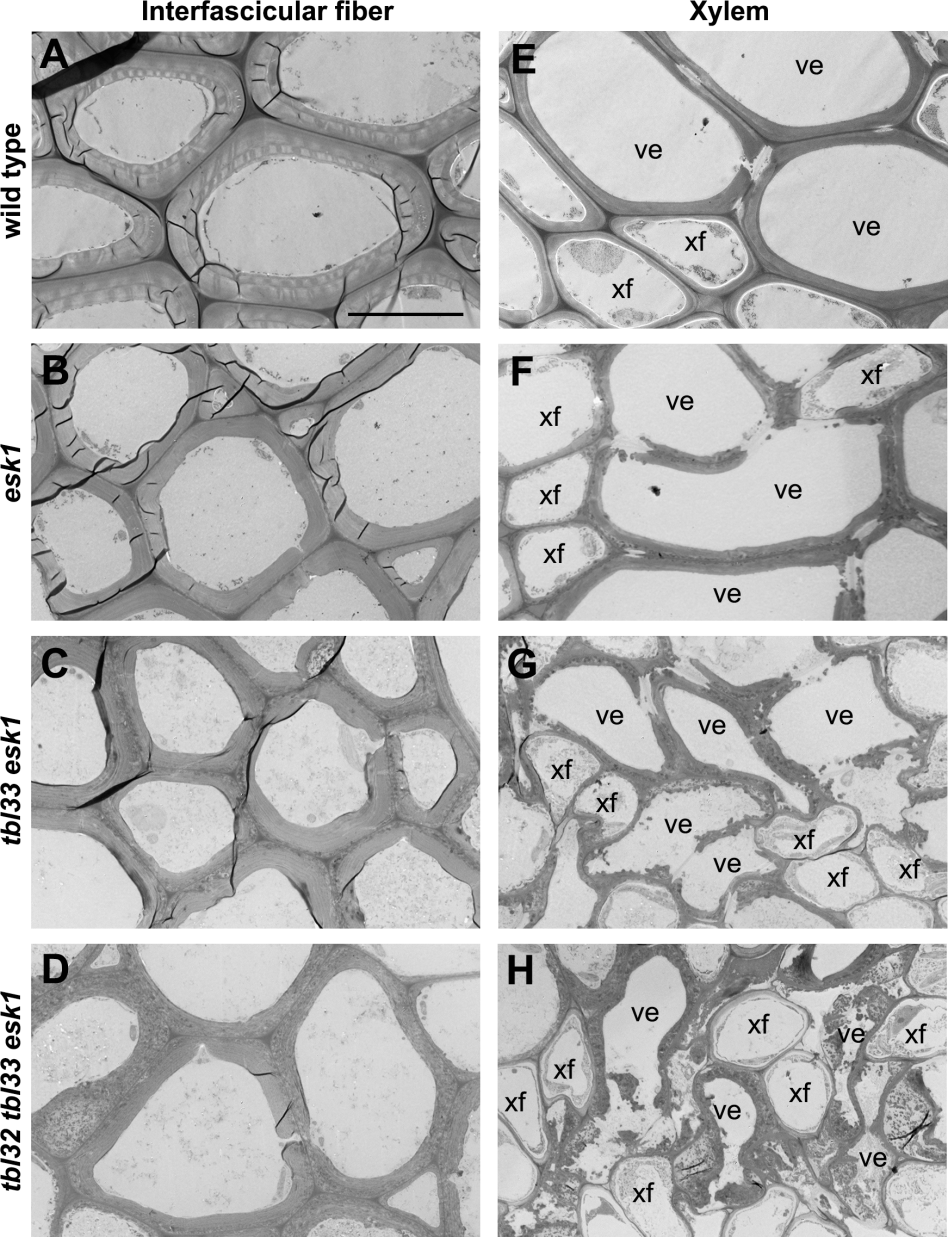
Transmission electron micrographs of interfascicular fibers and xylem in the wild type and various mutants. The bottom internodes of stems of 8-week-old wild-type and *esk1* plants, 12-week-old *tbl33 esk1* plants, and 16-week-old *tbl32 tbl33 esk1* plants were sectioned for visualization of walls of interfascicular fibers and xylem vessels. (A) to (D) Cross sections of interfascicular fibers showing defective secondary wall thickening in *esk1* (B), *tbl33 esk1* (C), and *tbl32 tbl33 esk1* (D) compared with the wild type (A). (E) to (H) Cross sections of xylem cells showing various degrees of deformation in vessels in *esk1* (F), *tbl33 esk1* (G), and *tbl32 tbl33 esk1* (H) compared with the wild type (E). ve, vessel; xf, xylary fiber. Bar in (A) = 10.5 μm for (A) to (H).

**Fig 6:**
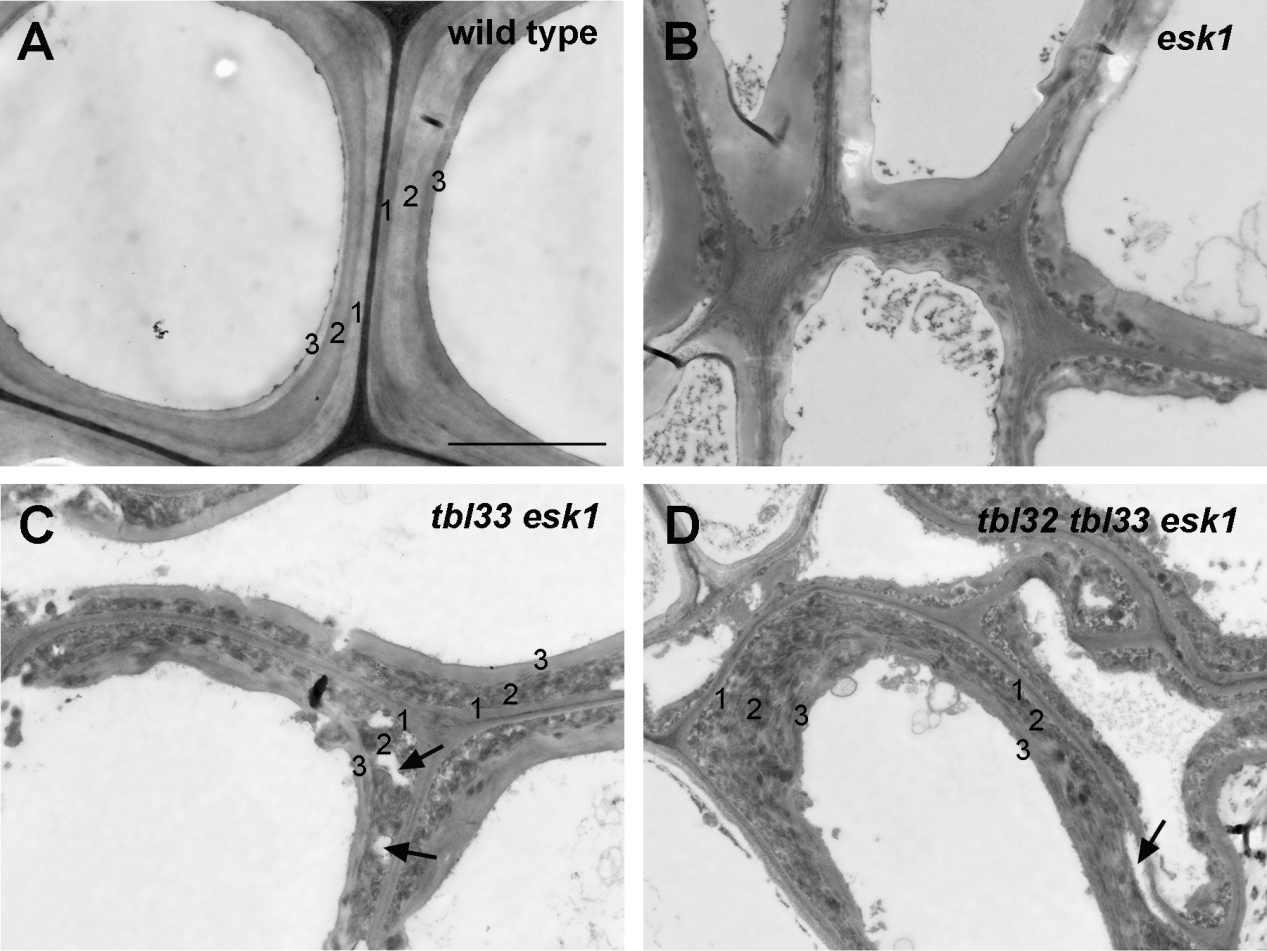
Effects of reduced acetylation of xylan on secondary wall structure in xylem vessels of *esk1* (B), *tbl33 esk1* (C) and *tbl32 tbl33 esk1* (D) compared with the wild type (A). Ultrathin stem sections were stained with lead citrate and uranyl acetate and visualized for vessel secondary wall structure under a transmission electron microscope. The numbers 1, 2 and 3 marked on vessel secondary walls denote the S1, S2 and S3 layers, respectively. Note the drastic alteration in the staining pattern of the S2 layer as well as disintegrated walls in the S2 layer (arrows) in *tbl33 esk1* (C) and *tbl32 tbl33 esk1* (D). Bar in (A) = 3 μm for (A) to (D).

**Fig 7:**
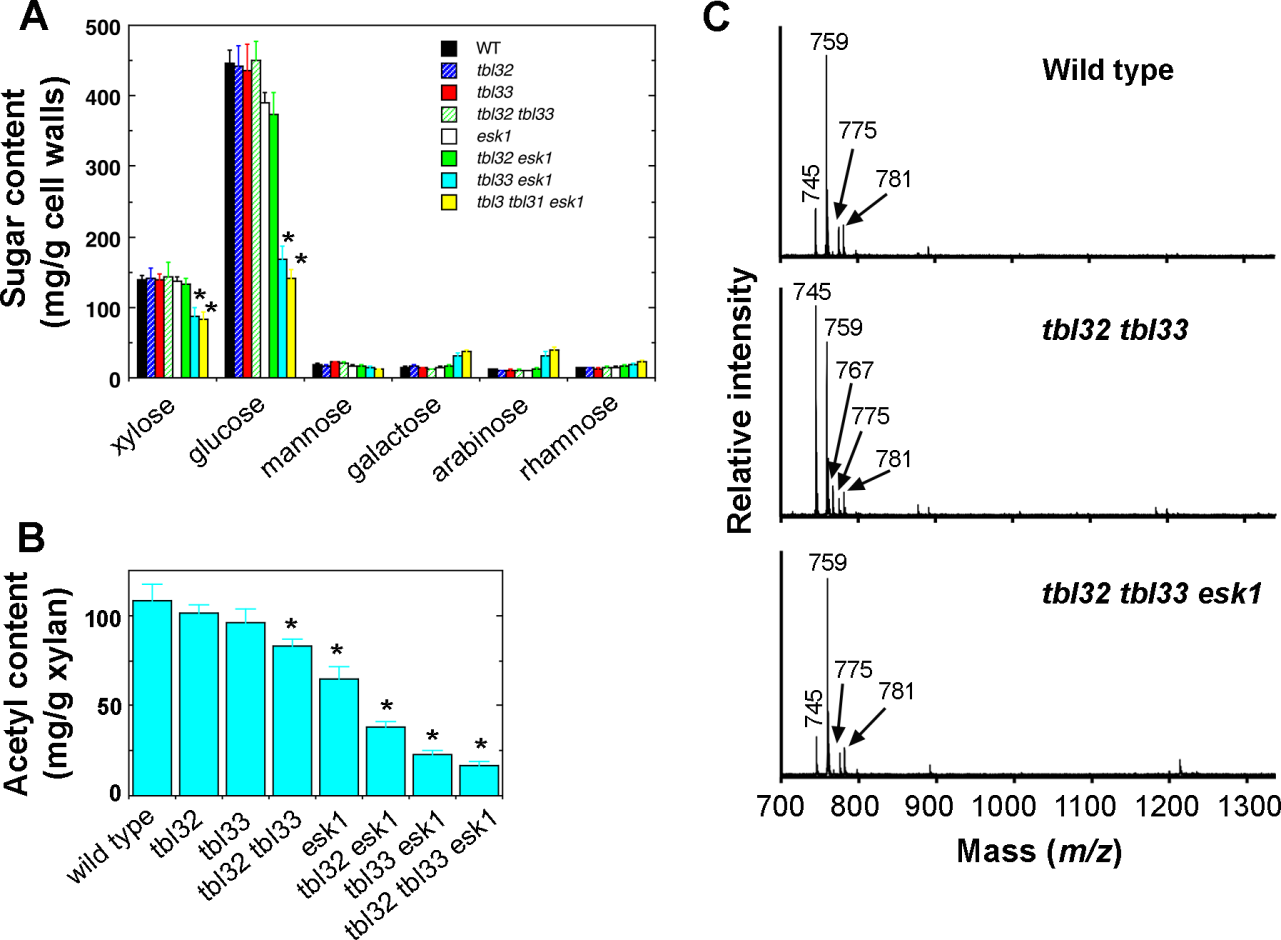
Measurement of cell wall sugar composition and acetyl contents in the wild type and various mutants. The inflorescence stems of 8-week-old wild type and *esk1* plants, 12-week-old *tbl33 esk1* plants, and 16-week-old *tbl32 tbl33 esk1* plants were used for extraction of cell wall residues and xylan. (A) Cell wall composition analysis revealed a reduction in the amounts of xylose and glucose in *tbl33 esk1* and *tbl32 tbl33 esk1* compared with the wild type and *esk1*. (B) Acetyl contents in DMSO-extracted xylans of the wild type and various mutants. Note the drastic reduction in the acetyl contents in *tbl32 esk1*, *tbl33 esk1*, and *tbl32 tbl33 esk1*. Error bars denote SD of the data from three separate pools of samples. Asterisks in (A) and (B) indicate statistically significant differences compared with the wild type (*p* < 0.001). (C) MALDI-TOF-MS analysis of xylooligomers generated by endoxylanase digestion of KOH-extracted xylan from the wild type (top panel), *tbl32 tbl33* (middle panel) and *tbl32 tbl33 esk1* (bottom panel). The ion peaks at *m/z* 745 and 759 are attributed to (GlcA)Xyl_4_ and (MeGlcA)Xyl_4_, respectively. Those at *m/z* 767 and 781 correspond to the disodiated species of (GlcA)Xyl_4_ and (MeGlcA)Xyl_4_, respectively. The ion at *m/z* 775 corresponds to (Gal-GlcA)Xyl_3_ [38].

Like several other xylan mutants [34–37], the *esk1* mutant had deformed xylem vessels, which was proposed to be the cause of reduced plant growth due to the impairment of water transport [3]. To find out whether the exacerbated growth defects observed in *tbl33 esk1* and *tbl32 tbl33 esk1* were correlated with the severity of defective xylem vessels, we examined their xylem morphology. While xylem vessels in stems and root-hypocotyls of the wild type were round or oval-shaped (Fig 4A and 4I) and some of those in *esk1* had mild deformation (Fig 4B and 4J), those in the stems and root-hypocotyls of *tbl33 esk1* were severely deformed (Fig 4C and 4K) and those in *tbl32 tbl33 esk1* appeared to have completely collapsed, leaving only a small space in each vessel (Fig 4D and 4L). Tranmission electron microscopy further revealed that vessels walls in *tbl33 esk1* and *tbl32 tbl33 esk1* appeared to have irregular shapes and some of them were disintegrated compared with those of the wild type and *esk1* (Fig 5E–5H). A close-up examination of vessel walls showed that while the wild type had three distinct secondary wall layers, namely S1, S2 and S3 (Fig 6A), such a layered deposition of secondary walls was not evident in *esk1* (Fig 6B). Interestingly, three secondary wall layers were still distinguishable in *tbl33 esk1* and *tbl32 tbl33 esk1*, but the staining pattern in the S2 layer was drastically altered and some areas of the S2 layer appeared to be torn apart (Fig 6C and 6D), indicating an alteration in secondary wall structure. In addition, the inner surface of secondary walls became uneven in the mutants (Fig 6B–6D) compared with the wild type (Fig 6A). Defective secondary wall thickening was also seen in the interfascicular fibers of *esk1*, *tbl33 esk1* and *tbl32 tbl33 esk1* (Figs 4E–4H and 5A–5D). Cell wall composition analysis showed that the amounts of xylose and glucose in the stem cell walls of both *tbl33 esk1* and *tbl32 tbl33 esk1* were greatly reduced (Fig 7A), indicating that the deposition of xylan and cellulose was affected. These results demonstrate that mutations of *TBL32* and *TBL33* greatly exacerbated the *esk1* mutant phenotypes, suggesting that they may play a similar role as *ESK1* in xylan acetylation.

### Simultaneous Mutations of *TBL32*, *TBL33* and *ESK1* Lead to a Reduction in the Frequency of Acetyl Substitutions in Xylan

To investigate whether TBL32 and TBL33 are involved in xylan acetylation, we measured the acetyl contents in xylans extracted from the single, double and triple mutants of *TBL32*, *TBL33*, and *ESK1*. Although the xylan acetyl contents in the *tbl32* and *tbl33* single mutants had no significant reduction, that in the *tbl32 tbl33* double mutant was decreased to 77% of that of the wild type (Fig 7B). Compared with *esk1* alone in which the acetyl content was reduced to 60% of that of the wild type, the acetyl contents in *tbl32 esk1* and *tbl33 esk1* were lowered to 35% and 21%, respectively, of that of the wild type. A further reduction in xylan acetyl content was seen in the *tbl32 tbl33 esk1* triple mutant, which was down to 15% of that of the wild type (Fig 7B).

**Fig 8:**
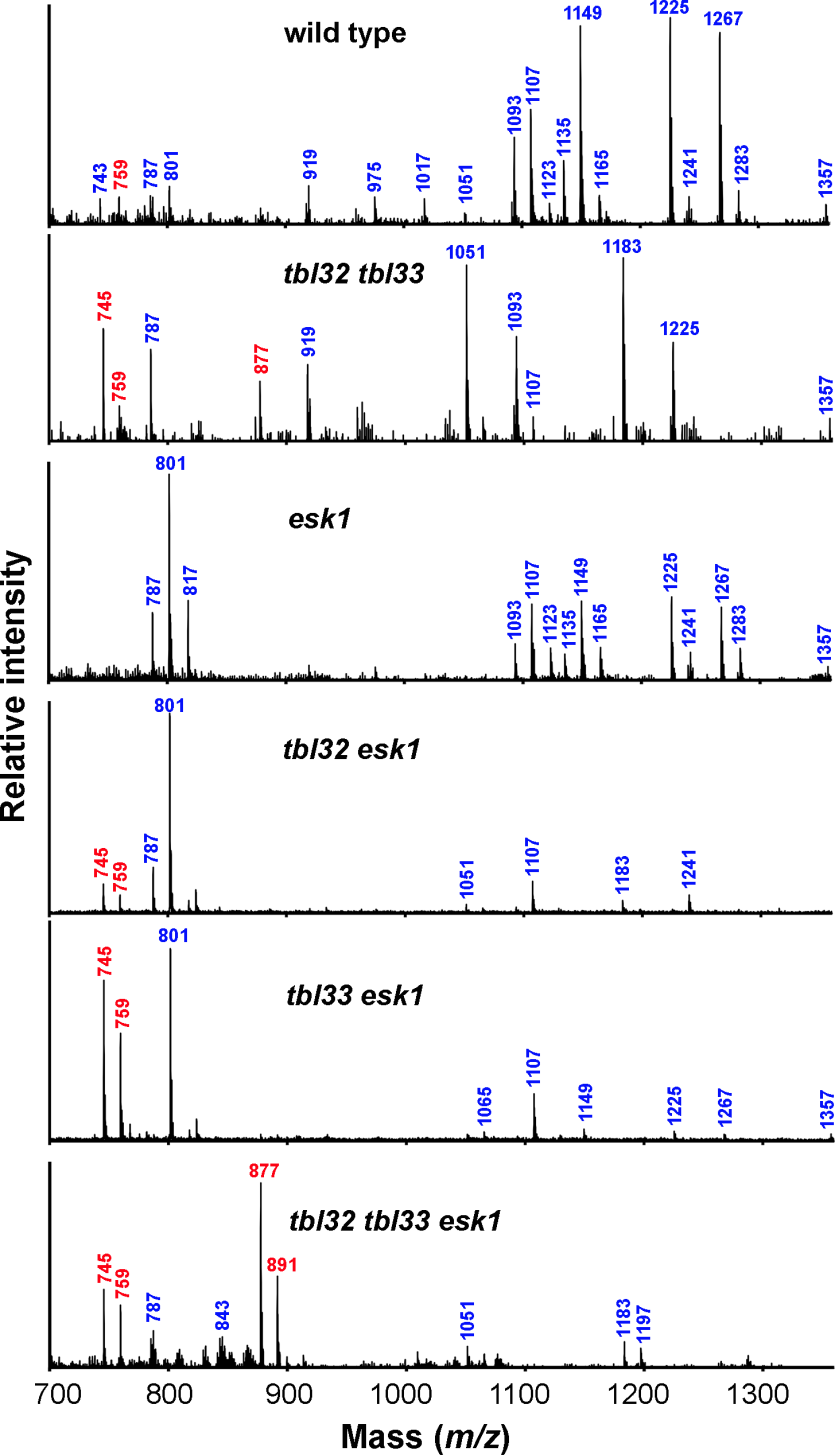
MALDI-TOF MS analysis of acetylated xylans of the wild type and various mutants. DMSO-extracted xylans were digested with endoxylanase and subject to MALDI-TOF MS. The major ion peaks of masses are indicated and their xylooligomer structures are listed below. Xyl_n_(GlcA)_n_(Ac)_n_ denotes a xylooligomer (with n number of xylosyl residues) substituted with n number of GlcA and n number of acetyl groups. *m/z* 743, Xyl_5_(Ac); *m/z* 745, Xyl_4_(GlcA); *m/z* 759, Xyl_4_(MeGlcA); *m/z* 787, Xyl_4_(GlcA)(Ac); *m/z* 801, Xyl_4_(MeGlcA)(Ac); *m/z* 817, Xyl_3_(MeGlcA)_2_; *m/z* 843, Xyl_4_(MeGlcA)(Ac)_2_; *m/z* 877, Xyl_5_(GlcA); *m/z* 891, Xyl_5_(MeGlcA); *m/z* 919, Xyl_5_(GlcA)(Ac); *m/z* 975, Xyl_5_(MeGlcA)(Ac)_2_; *m/z* 1017, Xyl_5_(MeGlcA)(Ac)_3_; *m/z* 1051, Xyl_6_(GlcA)(Ac); *m/z* 1065, Xyl_6_(MeGlcA)(Ac); *m/z* 1093; Xyl_6_(GlcA)(Ac)_2_; *m/z* 1107; Xyl_6_(MeGlcA)(Ac)_2_; *m/z* 1123, Xyl_5_(MeGlcA)_2_(Ac); *m/z* 1135, Xyl_6_(GlcA)(Ac)_3_; *m/z* 1149, Xyl_6_(MeGlcA)(Ac)_3_; *m/z* 1165, Xyl_5_(MeGlcA)_2_(Ac)_2_; *m/z* 1183, Xyl_7_(GlcA)(Ac); *m/z* 1197, Xyl_7_(MeGlcA)(Ac); *m/z* 1225, Xyl_7_(GlcA)(Ac)_2_; *m/z* 1241, Xyl_6_(GlcA)(MeGlcA)(Ac); *m/z* 1267, Xyl_7_(GlcA)(Ac)_3_; *m/z* 1283, Xyl_6_(GlcA)(MeGlcA)(Ac)_2_; *m/z* 1357, Xyl_8_(GlcA)(Ac)_2_. Note the increase in the abundance of ions at *m/z* 745, 759, 877, and 891 (marked in red) corresponding to non-acetylated xylooligomers in *tbl32 tbl33*, *tbl32 esk1*, *tbl33 esk1*, and *tbl32 tbl33 esk1*.

The finding that the double and triple mutants of *TBL32*, *TBL33*, and *ESK1* exhibited a drastic reduction in xylan acetyl content prompted us to examine the effects of their mutations on acetyl distribution patterns in xylan. Xyloligosaccharides were generated from DMSO-extracted xylans with endoxylanase and were subsequently analyzed using matrix-assisted laser desorption ionization-time-of-flight mass spectrometry (MALDI-TOF-MS) (Fig 8). Wild-type xylooligosaccharides displayed predominant signal peaks corresponding to Xyl_6_(GlcA)(Ac)_2_, Xyl_6_(MeGlcA)(Ac)_2_, Xyl_6_(MeGlcA)(Ac)_3_, Xyl_7_(GlcA)(Ac)_2_, and Xyl_7_(GlcA)(Ac)_3_ at *m/z* 1093, 1107, 1149, 1225, and 1267, respectively. These abundant xylooligomers contain two to three acetyl groups. Also present were some less abundant signal peaks, which correspond to Xyl_4_ to Xyl_8_ containing various numbers of acetyl groups, GlcA and MeGlcA residues. The pattern of signal peaks exhibited by xylooligosaccharides from *tbl32 tbl33* appeared to be distinct from that of wild-type xylooligosaccharides (Fig 8). While the signal peaks for Xyl_6_(MeGlcA)(Ac)_2_ at *m/z* 1107, Xyl_6_(MeGlcA)(Ac)_3_ at *m/z* 1149, and Xyl_7_(GlcA)(Ac)_3_ at *m/z* 1267 were severely diminished, the signal peaks for Xyl_6_(GlcA)(Ac) at *m/z* 1051 and Xyl_7_(GlcA)(Ac) at *m/z* 1183, both of which contain only one acetyl group, were drastically increased. More significantly, two signal peaks for non-acetylated Xyl_4_(GlcA) and Xyl_5_(GlcA) at *m/z* 745 and 877, respectively, were prominent in *tbl32 tbl33*. This alteration in xylan acetyl substitutions differed from that in *esk1*, which lacked the signal peaks for non-acetylated Xyl_4_(GlcA) at *m/z* 745 and Xyl_5_(GlcA) at *m/z* 877 but had the most abundant signal peak for Xyl_4_(MeGlcA)(Ac) at *m/z* 801 (Fig 8). These results show that the *tbl32 tbl33* mutations and the *esk1* mutation have different effects on acetyl group distribution patterns in xylan.

MALDI-TOF-MS analysis of xylooligomers from *tbl32 esk1* and *tbl33 esk1* revealed that compared with *esk1* alone, both double mutants exhibited a higher reduction in the signals peaks for longer xylooligosaccharides with two or more acetyl groups although they retained the most abundant signal peak for Xyl_4_(MeGlcA)(Ac) at *m/z* 801 (Fig 8). In addition, the signal peaks for non-acetylated Xyl_4_(GlcA) and Xyl_4_(MeGlcA) at *m/z* 745 and 759, respectively, were prominent in *tbl33 esk1* and also present in *tbl32 esk1* albeit in a lesser amount, which is consistent with the xylan acetyl content measurement showing a higher reduction in *tbl33 esk1* than *tbl32 esk1* (Fig 7B). The most dramatic alteration was seen in the *tbl32 tbl33 esk1* triple mutant in which the signal peaks corresponding to acetylated xylooligomers were greatly diminished and the most prominent signal peaks were *m/z* 745, 759, 877, and 891, which correspond to non-acetylated Xyl_4_(GlcA), Xyl_4_(MeGlcA), Xyl_5_(GlcA), and Xyl_5_(MeGlcA), respectively (Fig 8). MALDI-TOF-MS analysis of xylooligomers released from xylanase digestion of KOH-extracted xylans showed predominant signal peaks at *m/z* 745 and 759 for Xyl_4_(GlcA) and Xyl_4_(MeGlcA), respectively, in both wild type and *tbl32 tbl33 esk1* (Fig 7C), indicating that xylanase digestion efficiency is the same for both wild-type and *tbl32 tbl33 esk1* xylans when acetyl groups are removed. Therefore, the observed appearance of ion peaks for Xyl_5_(GlcA) and Xyl_5_(MeGlcA) in xylooligomers released from xylanase digestion of *tbl32 tbl33 esk1* acetylated xylan is likely caused by an altered acetylation degree, which may affect xylanase digestion efficiency. These results demonstrate that endoxylanase digestion of acetylated xylans from the double and triple mutants of *TBL32*, *TBL33*, and *ESK1* leads to generation of xylooligosaccharides with fewer or no acetyl groups compared with the wild type, suggesting that the mutant xylans have a significant reduction in the frequency of acetyl substitutions.

### Loss of 3-*O*-Acetylation of 2-*O*-GlcA-Substituted Xylosyl Residues in Xylan of the *tbl32 tbl33* Mutant

**Fig 9:**
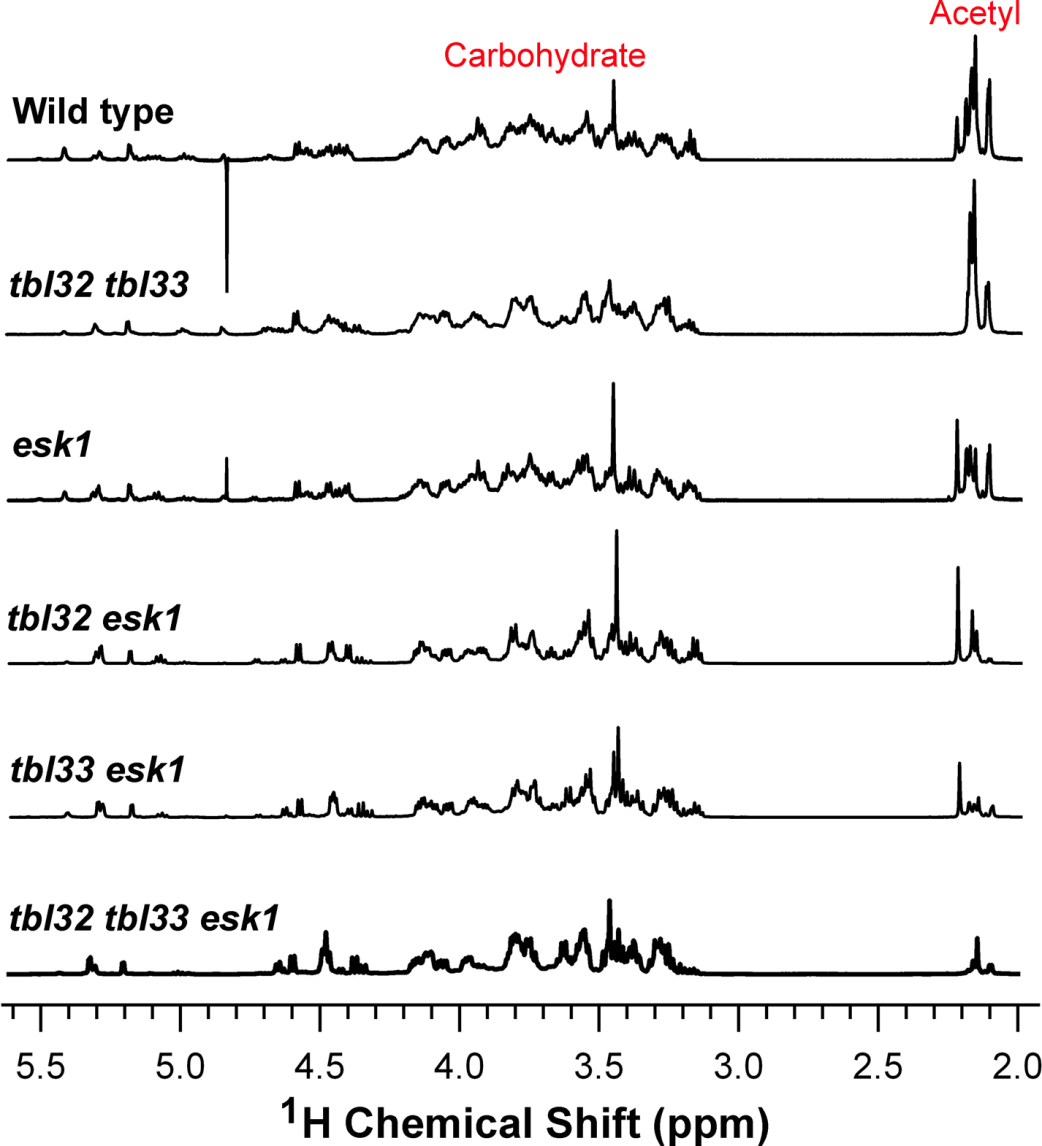
^1^H NMR spectra of acetylated xylans from the wild type, *tbl32 tbl33*, *esk1*, *tbl32 esk1*, *tbl33 esk1*, and *tbl32 tbl33 esk1*. The resonance regions corresponding to acetyl groups and carbohydrate are indicated.

To found out what specific changes in acetyl substitutions were resulted from the mutations, we examined acetyl substitutions in xylans from the double and triple mutants of *TBL32*, *TBL33*, and *ESK1* using nuclear magnetic resonance (NMR) spectroscopy. Wild-type xylan exhibited resonances around 2.2 ppm for acetyl groups and between 3.0 and 5.3 ppm for sugar groups (Fig 9) [13]. Although xylans of various mutants had the resonances for acetyl groups, integration analysis showed that the relative amounts of acetyl groups in these mutants were reduced compared with the wild type (Table 1), consistent with the xylan acetyl content measurement (Fig 7B). The most drastic reduction was seen in *tbl32 tbl33 esk1* in which the average degree of xylan acetyl substitutions (DS_Ac_) was reduced to 0.12 compared with 0.6 in the wild type (Table 1).

**Table 1:** Relative integrated values of acetyl groups and carbohydrates in DMSO-extracted xylans from the wild type and various mutants.

Sample	Carbohydrates^a^	Acetyl groups^b^	DS_AC_	Relative amount of acetyl groups^c^
Wild type	1	0.19	0.60	100
*tbl32 tbl33*	1	0.17	0.53	88
*esk1*	1	0.14 *	0.44	73
*tbl32 esk1*	1	0.10 *	0.31	52
*tbl33 esk1*	1	0.07 *	0.22	37
*tbl32 tbl33 esk1*	1	0.04 *	0.12	20

^a^ The integration of carbohydrates was calculated from the NMR resonance between 3 and 5.5 ppm (Fig 9) and taken as 1.

^b^ The integration of acetyl groups was determined from the ratio of the NMR resonance of acetyl groups to that of carbohydrates.

^c^ The amount of acetyl groups in the wild-type xylan is taken as 100, and the relative amount of acetyl groups in the mutants is calculated from the ratio of acetyl groups in the mutants over that of the wild type.

The integration data are from the average of three NMR spectra for each sample and asterisks indicate statistically significant differences between the wild type and the mutants.

DS_AC_, degree of substitution of xylosyl residues by acetyl groups.

**Fig 10:**
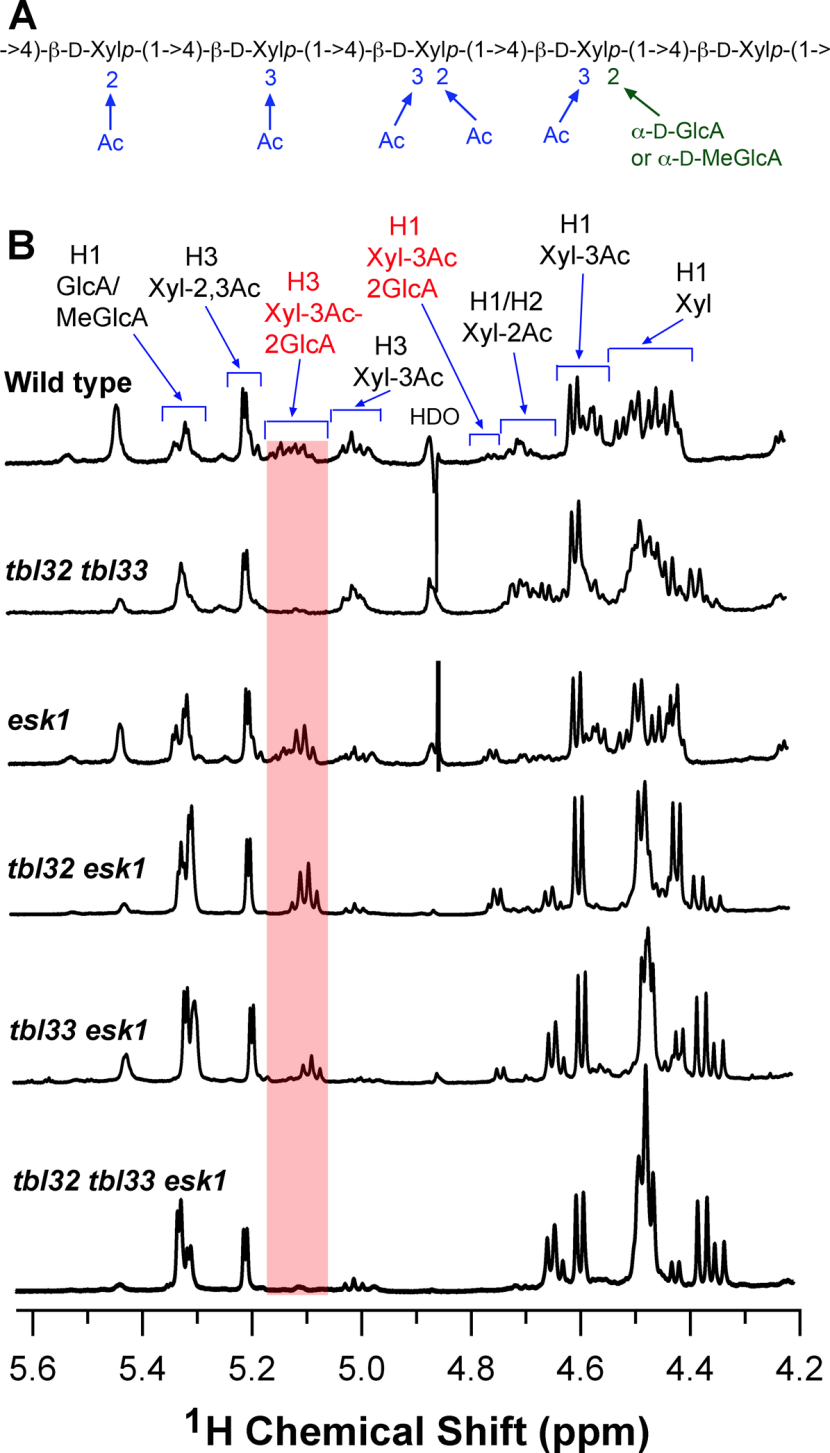
Distribution patterns of xylan acetyl substitutions in the wild type and various mutants. (A) Diagram of an acetylated xylooligomer from wild-type Arabidopsis xylan. (B) The fingerprint regions of the ^1^H NMR spectra of acetylated xylans from the wild type, *tbl32 tbl33*, *esk1*, *tbl32 esk1*, *tbl33 esk1*, and *tbl32 tbl33 esk1*. The resonances for non-acetylated (Xyl), 2-*O*-acetylated (Xyl-2Ac), 3-*O*-acetylated (Xyl-3Ac), 2,3-di-*O*-acetylated (Xyl-2,3Ac), 3-*O*-acetylated 2-*O*-GlcA-substituted xylosyl residues (Xyl-3Ac-2GlcA) and GlcA/MeGlcA are labeled. Note the loss of the resonances of Xyl-3Ac-2GlcA in *tbl32 tbl33* and *tbl32 tbl33 esk1*.

We next investigated whether the reduction in xylan acetyl substitutions was regiospecific by examining the fingerprint region of the ^1^H NMR spetra of xylan. The assignment of proton resonances for different acetyl substituions was based on published data for xylans from aspen and Arabidopsis [12,38]. The proton resonances for 2-*O*-acetylated (Xyl-2Ac), 3-*O*-acetylated (Xyl-3Ac), 2,3-di-*O*-acetylated (Xyl-2,3Ac), and 3-*O*-monoacetylated, 2-*O*-GlcA-substituted xylosyl residues (Xyl-3Ac-2GlcA) were evident in the wild-type xylan (Fig 10A and 10B). Examination of the resonances corresponding to xylosyl residues with various acetyl substitutions in *tbl32 tbl33* xylan showed a nearly complete loss of the resonances for Xyl-3Ac-2GlcA (Fig 10B). Integration analysis revealed that the resonance abundance for Xyl-3Ac-2GlcA in *tbl32 tbl33* was reduced to 4% of that of the wild type (Table 2). In addition, a reduction in the resonances for Xyl-3Ac and Xyl-2,3Ac was also observed in *tbl32 tbl33*. In contrast, the resonance signals for Xyl-2Ac were increased significantly in *tbl32 tbl33* (Table 2). These results demonstrate that mutations of *TBL32* and *TBL33* result in a regiospecific defect in xylan acetyl substitutions that is different from the *esk1* mutation, which specidfically reduces xylan 2-*O*- and 3-*O*-monoacetylation (Fig 10B) [3].

**Table 2:** Relative content of acetyl groups in xylans from the wild type and various mutants.

Structural fragment	Relative abundance (mol%)					
	Wild type	*tbl32 tbl33*	*esk1*	*tbl32 esk1*	*tbl33 esk1*	*tbl32 tbl33 esk1*
Non-acetylated xylose (Xyl)	40	43	56 *	69 *	78 *	88 *
2-*O*-Acetylated xylose (Xyl-2Ac)	18	33 *	10 *	11 *	11 *	7 *
3-*O*-Acetylated xylose (Xyl-3Ac)	18	12 *	10 *	4 *	2 *	2 *
2,3-di-*O*-Acetylated xylose (Xyl-2,3Ac)	16	12 *	15	9 *	6 *	3 *
3-*O*-Acetylated, 2-*O*-GlcA-substituted xylose (Xyl-3Ac-2GlcA)	8	0.3 *	9	7	3 *	0.2 *
GlcA/MeGlcA	8	7	14 *	17 *	16 *	15 *

Non-acetylated and 2-*O*-monoacetylated xylosyl residues are integrated based on their anomeric proton resonances, and 3-*O*-monoacetylated, 2,3-di-*O*-acetylated and 3-*O*-acetylated 2-*O*- GlcA/MeGlcA-substituted xylosyl residues are integrated based on their H3 resonances (Fig 10). The total balance of acetyl groups was verified by integration of acetyl signals at 2.2 ppm (Fig 9). The integration data are from the average of three NMR spectra for each sample and asterisks indicate statistically significant differences between the wild type and the mutants.

The fingerprint region of the ^1^H NMR spetra of xylan from other mutants was also analyzed. Although the signal abundance for Xyl-2Ac in *tbl32 esk1* and *tbl33 esk1* was similar to that in *esk1* (Table 2), both mutants exhibited a more drastic reduction in the resonances for Xyl-3Ac compared with *esk1* (Fig 10). Integration analysis showed that the resonance abundance for Xyl-3Ac in *tbl32 esk1* and *tbl33 esk1* was reduced to 19% and 11%, respectively, of that of the wild type (Table 2). This was in contrast to 56% of that of the wild type in *esk1*, indicating redundant roles of *TBL32*, *TBL33* and *ESK1* in xylan 3-*O*-monoacetylation. In addition, a reduction in Xyl-3Ac-2GlcA was observed in *tbl33 esk1* and a reduction in Xyl-2,3Ac was seen in both *tbl32 esk1* and *tbl33 esk1* (Table 2). Like *tbl32 tbl33*, the *tbl32 tbl33 esk1* triple mutant had a nearly complete loss of the resonance signals for Xyl-3Ac-2GlcA. A higher level of decrease in the resonances for 3-*O*-monoacetylated and 2,3-di-*O*-acetylated xylosyl residues was evident in *tbl32 tbl33 esk1* compared with *tbl32 tbl33* (Fig 10; Table 2), suggesting that the *esk1* mutation exacerbates the effects of *tbl32 tbl33* mutations on xylan 3-*O*-monoacetylation and 2,3-di-*O*-acetylation. It was noted that the resonances for GlcA/MeGlcA were elevated in *esk1*, *tbl32 esk1*, *tbl33 esk1*, and *tbl32 tbl33 esk1* compared with the wild type (Fig 10; Table 2). This elevation in GlcA/MeGlcA substitutions is likely caused by the reduction in *O*-2 acetylation at xylosyl residues by the *esk1* mutation, which makes additional *O*-2 positions open for GlcA/MeGlcA substitutions [3]. This possibility was in agreement with the observation that the *esk1* mutation resulted in an increase in the MeGlcA level [39]. In contrast, the *gux1/2/3* xylan, which lacked GlcA substitutions [40], had an elevated level of 2-*O*-acetylation [41]. Together, these findings indicate that TBL32 and TBL33 play a dominant role in mediating 3-*O*-acetylation of 2-*O*-GlcA-substituted xylosyl residues and are also involved in xylan 3-*O*-monoacetylation and 2,3-di-*O*-acetylation.

## Discussion

One of the major polymers in lignocellulosic biomass is acetylated xylan, which contributes to the recalcitrance of biomass for biofuel production [42]. Therefore, understanding how xylan is acetylated is not only important in broadening our knowledge in basic plant biology but also has important implications in genetic engineering of biomass tailored for biofuel production. Several proteins, including RWAs, AXY9, and ESK1, have been implicated in xylan acetylation. While RWAs and AXY9 have been proposed to be involved in providing the supply of acetyl donors for acetylation of xylan and other wall polysaccharides [24–26], ESK1 is an acetyltransferase mediating xylan 2-*O*- and 3-*O*-monoacetylation [3,4,30]. However, it remains unknown what genes are responsible for xylan 2,3-di-*O*-acetylation and 3-*O*-acetylation of 2-*O*-GlcA-substituted xylosyl residues [3]. Our finding that mutations of *TBL32* and *TBL33* cause a loss of acetyl substitutions at *O*-3 of 2-*O*-GlcA-substituted xylosyl residues in xylan increases our understanding of the biochemical process of xylan acetylation.

### TBL32 and TBL33 Are Putative Acetyltransferases Mediating Acetyl Substitutions at *O*-3 of 2-*O*-GlcA-Substituted Xylosyl Residues in Xylan

Several lines of molecular and genetic evidence indicate the essential roles of TBL32 and TBL33 in xylan *O*-acetylation. First, both TBL32 and TBL33 are localized in the Golgi, where xylan is synthesized. Second, simultaneous mutations of *TBL32* and *TBL33* cause a significant reduction in xylan acetylation. In particular, structural analysis of xylan has led to the discovery that simultaneous mutations of *TBL32* and *TBL33* cause a nearly complete loss of acetyl substitutions at *O*-3 of 2-*O*-GlcA-substituted xylosyl residues in xylan, which is in sharp contrast to the *esk1* mutation affecting xylan 2-*O*- and 3-*O*-monoacetylation [3]. We propose that TBL32 and TBL33 are acetyltransferases mediating acetyl substitutions at *O*-3 of 2-*O*-GlcA-substituted xylosyl residues. The fact that the *tbl32 tbl33* mutant has a nearly complete loss of acetyl substitutions at *O*-3 of 2-*O*-GlcA-substituted xylosyl residues implies that xylosyl residues are first substituted with GlcA at *O*-2 and then with acetyl groups at *O*-3. This hypothesis is in congruent with the observation that xylan in the *tbl32 tbl33* mutant still remains acetyl groups at *O*-3 of xylosyl residues without GlcA substitutions at *O*-2.

The finding that TBL32 and TBL33 differ from ESK1 in mediating xylan acetyl substitutions provides further evidence supporting the hypothesis that different acetyltransferase activities mediate the acetylation at different positions of xylosyl residues [3]. It may also have important implications in studying the biochemical mechanisms underlying acetylation of other cell wall polysaccharides. Because xyloglucans and pectins from some species can be both mono- and di-acetylated [10,11,20,21], it is envisaged that different acetyltransferase activities may also be involved in catalyzing the mono- and di-acetylation of those polysaccharides. It should be pointed out that although *TBL32* does not exhibit a predominant expression in stems nor is it regulated by SND1, the fact that simultaneous mutations of *TBL32* and *TBL33* cause an additive effect on xylan acetylation indicates that both of them are involved in xylan acetylation during secondary wall biosynthesis. However, the expression level of *TBL32* is much lower than that of *TBL33*, indicating that TBL32 might play a minor role in xylan acetylation. In addition, *TBL32* transcripts were detected in pith parenchymatous cells, suggesting that it may also be involved in xylan acetylation in primary cell walls.

The loss of acetyl substitutions at *O*-3 of 2-*O*-GlcA-substituted xylosyl residues in *tbl32 tbl33* xylan is consistent with the generation of non-acetylated xylooligomers, Xyl_4_(GlcA) and Xyl_5_(GlcA), from its digestion by endoxylanase. Although the *esk1* mutation causes a much higher overall reduction in acetyl content than *tbl32 tbl33*, neither Xyl_4_(GlcA) nor Xyl_5_(GlcA) is evident in the xylooligomers generated from xylanase digestion of *esk1* xylan. Instead, a predominant xylooligomer generated from digestion of *esk1* xylan is Xyl_4_(MeGlcA)(Ac), in which the acetyl group is most likely attached to *O*-3 of xylosyl residues substituted at *O*-2 with MeGlcA because the *esk1* mutation only reduces the levels of Xyl-2Ac and Xyl-3Ac but not Xyl-3Ac-2GlcA. Strikingly, the vast majority of xylooligomers generated from xylanase digestion of *tbl32 tbl33 esk1* xylan are non-acetylated xylooligomers, Xyl_4_(GlcA/MeGlcA) and Xyl_5_(GlcA/MeGlcA), indicating that TBL32 and TBL33 together with ESK1 are responsible for the majority of xylan acetylation in Arabidopsis stems. This conclusion is supported by the fact that the acetyl content in *tbl32 tbl33 esk1* xylan is only 15% of that in the wild type.

TBL32 and TBL33 join the list of DUF231 proteins known to be involved in *O*-acetylation of cell wall polymers. Among the 46 members of Arabidopsis DUF231 family [27], three of them, AXY4, AXY4L, and ESK1, have previously been proven to mediate the acetylation of xyloglucan or xylan [3,4,28]. Considering the fact that several other wall polymers, including glucomannan, pectins, and lignin, are also acetylated [1], it is possible that other members of the DUF231 family confer acetyltransferase activities involved in their acetylation. Functional characterization of other DUF231 members will likely increase our knowledge of the biochemical mechanisms controlling the acetylation of other wall polymers.

### TBL32 and TBL33 Are Also Involved in 3-*O*-Monoacetylation and 2,3-di-*O*-Acetylation of Xylosyl Residues in Xylan

Structural analysis of *tbl32 tbl33* xylan has also revealed defects in 3-*O*-monoacetylation and 2,3-di-*O*-acetylation of xylosyl residues. The reduction in 3-*O*-monoacetylated xylosyl residues is most prominent in *tbl32 tbl33 esk1* in which only about 11% of the wild-type level remains, indicating that TBL32, TBL33, and ESK1 function redundantly and are the major players in mediating 3-*O*-monoacetylation of xylosyl residues. In addition, 2,3-di-*O*-acetylation in *tbl32 tbl33 esk1* xylan is reduced to 19% of that of the wild type. It is currently unknown how 2,3-di-*O*-acetylation of xylosyl residues in xylan is carried out; a xylosyl residue can be acetylated at *O*-2 first and then at *O*-3 or vice versa. It is intriguing to note that *tbl32 tbl33* xylan exhibits a significant increase in the relative level of 2-*O*-monoacetylated xylosyl residues. One logical explanation is that TBL32 and TBL33 are also involved in xylan 2,3-di-*O*-acetylation by mediating the addition of acetyl groups at *O*-3 of xylosyl residues already substituted at *O*-2 with acetyl groups, and therefore, their mutations result in a decrease in 2,3-di-*O*-acetylation and a concomitant increase in 2-*O*-monoacetylation of xylosyl residues. This increase in 2-*O*-monoacetylated xylosyl residues is diminished in *tbl32 tbl33 esk1*, which is consistent with the role of ESK1 in catalyzing 2-*O*-monoacetylation of xylosyl residues. Our findings provide genetic evidence indicating that xylan 2,3-di-O-acetylation is likely mediated by two different acetyltransferase activities; one, such as ESK1, first adds acetyl groups at *O*-2 of xylosyl residues and the other, such as TBL32 and TBL33, subsequently adds acetyl groups at *O*-3 of these xylosyl residues.

It should be cautioned that acetyl groups may migrate between *O*-2 and *O*-3 positions of xylosyl residues *in vivo* and *in vitro*, and thus it is possible that the observed regiospecific defect and abundance in xylan acetylation in the mutants could be caused by spontaneous migration of acetyl groups. However, this possibility is unlikely based on the following lines of evidence. First, the extraction method for acetylated xylan used in this study is the same one as reported by Evtuguin et al. [14], which verified that the abundance and positions of acetyl groups are not altered under the conditions used for xylan extraction. A report that acetyl groups at *O*-3 of xylosyl residues are not added by enzymatic reaction but rather migrated from those from *O*-2 is based on the *in vitro* enzymatic assay showing that 3-*O*-acetylation occurs slower than 2-*O*-acetylation [30], but no direct proof is provided for this conclusion. Second, we used the same conditions to extract xylans from the wild type and *esk1*, but these isolated xylans did not show such a regiospecific acetylation defect, i.e., a lack of 3-*O*-acetylation of 2-*O*-GlcA-substituted xylosyl residues, which argues against the possibility that random migration of acetyl groups causes the observed regiospecific acetyl defects in the mutants. Third, we have recently shown that mutations of two other ESK1 close homologs, *TBL3* and *TBL31*, result in a specific reduction in 3-*O*-acetylation of xylan [39], indicating that acetyl groups at *O*-3 of xylosyl residues are not simply migrated from those from *O*-2. Fourth, we used the same conditions to extract xylans from the wild type and various mutants, but xylan acetyl content from the *tbl32 tbl33 esk1* mutant has only about 15% of that in the wild type, indicating that this drastic reduction of acetyl abundance could not be simply caused by spontaneous migration leading to a loss of acetyl groups. The available evidence indicates that the regiospecific acetylation defects in mutant xylan, i.e., the loss of 3-*O*-acetylation of 2-*O*-GlcA-substituted xylosyl residues and reductions in 3-*O*-monoacetylation and 2,3-di-*O*-acetylation of xylosyl residues, are attributed to the defects in acetyltransferase activities catalyzing regiospecific acetylation rather than random acetyl migration. The degree of spontaneous acetyl group migration in xylan and its contribution to acetyl substitution patterns of xylan *in vivo* remain to be investigated.

### Xylan Acetylation Is Essential for Normal Plant Growth and Secondary Wall Deposition

The double and triple mutants of *TBL32*, *TBL33*, and *ESK1* exhibit a varied degree of reduction in acetyl groups in xylan, which is correlated with the severity of defects in plant growth and development. The *tbl32 tbl33* double mutant has a xylan acetyl content of about 77% of that of the wild type and no apparent defects in plant growth, indicating that a mild reduction in xylan acetylation does not have any negative impacts on plant growth. Both *esk1* and *tbl32 esk1*, whose xylan acetyl content is reduced to about 60% and 35% of that of the wild type, respectively, exhibit reduced plant growth. In contrast, the growth of *tbl33 esk1* and *tbl32 tbl33 esk1*, whose xylan acetyl content is decreased to about 21% and 15% of that of the wild type, respectively, is so severely retarded that only small inflorescences are produced after prolonged growth. The severity of defects in plant growth is also correlated with the degree of severity of xylem vessel deformation. The xylem vessels in both *tbl33 esk1* and *tbl32 tbl33 esk1* are so severely collapsed that only a small slit remains in each vessel. The severe deformation of xylem vessels most likely impedes water transport, thus leading to retardation of plant growth. This scenario resembles the simultaneous mutations of GT43 genes required for xylan backbone elongation, IRX9 and I9H or IRX9 and IRX14, in which the xylem vessels are severely collapsed and the plant growth is stunted [43].

The severe deformation of xylem vessels in *tbl33 esk1* and *tbl32 tbl33 esk1* are most likely caused by defects in secondary wall deposition. The amounts of both xylose and glucose, the main sugars for xylan and cellulose, respectively, are significantly reduced in these mutants. While the xylose amount is decreased to 60–62% of that of the wild type, the glucose amount is reduced to 32–38% of that of the wild type, indicating that cellulose deposition is more severely affected than xylan deposition in these mutants. Similarly, *esk1* and *tbl32 esk1* have a mild reduction in glucose content but no decrease in xylose content. Because acetyl substitutions of xylan affect the physical properties of xylan and its interaction with cellulose [44,45], it is likely that a severe reduction in xylan acetylation may alter the normal assembly of xylan and cellulose, which, in turn, leads to impeded biosynthesis and deposition of cellulose. This hypothesis is in congruent with the observation that the S2 layer of vessel secondary walls showed a drastically altered staining pattern in the *tbl33 esk1* and *tbl32 tbl33 esk1* mutants with a severe reduction in xylan acetylation. Because the layered secondary walls are the result of the oriented deposition of cellulose microfibrils [46], such an alteration in the S2 layer staining pattern implies a change in cellulose microfibril deposition. The alteration in cellulose microfibril assembly in the S2 layer may weaken the wall strength to resist the negative pressure generated during transpiration, resulting in disintegration of the S2 layer as observed in the mutants. It should be noted that the thickness of vessel secondary walls in the mutants is not significantly reduced compared with that in the wild type, which further suggests that the severely collapsed vessels in the mutants are due to weakened wall strength rather than reduced wall thickness. This finding provides the first line of genetic evidence indicating the importance of xylan acetylation in normal assembly of secondary wall polymers and hence secondary wall strength. It should be pointed out that a complete loss of xylan GlcA substitutions only has a mild impact on secondary wall deposition and strength and plant growth [40], implying that xylan GlcA substitutions play a much less important role in the assembly of secondary wall polymers than xylan acetylation.

We have demonstrated a role of TBL32 and TBL33, two ESK1 close homologs, in 3-*O*-acetylation of 2-*O*-GlcA-substituted xylosyl residues in xylan. TBL32 and TBL33 are also involved in 3-*O*-monoacetylation and 2,3-di-*O*-acetylation of xylosyl residues. Our genetic analysis indicates that 3-*O*-acetylation of 2-*O*-GlcA-substituted xylosyl residues in xylan is mediated by first addition of GlcA at *O*-2 and then acetylation at *O*-3 and that xylan 2,3-di-*O*-acetylation is most likely carried out by addition of acetyl groups first at *O*-2 and subsequently at *O*-3. Our findings support the hypothesis that different acetyltransferase activities might mediate xylan acetyl substitutions at different positions, which enriches our understanding of the underlying biochemical mechanisms regarding the acetylation of wall polysaccharides in general.

## Materials and Methods

### Plant Materials

*Arabidopsis thaliana* plants (ecotype Columbia) were grown under 14-h-light/10-h-dark cycles in a growth room. Wild type and mutant plants were grown at the same time and under the same growth conditions for examination of their morphology and for collections of stem materials. At least three separate pools of plant materials were used for all the experimental analyses unless otherwise indicated.

### Gene Expression Analysis

Total RNA from various Arabidopsis organs was isolated with a Qiagen RNA isolation kit (Qiagen). Wild-type leaves and roots were from 6-week-old plants. Wild-type stems from 6-week-old plants were divided into top, middle and bottom parts, which represent the rapidly elongating internodes, internodes near cessation of elongation, and non-elongating internodes, respectively. For SND1 overexpressors, leaves from 4-week-old plants were used. For the *snd1 nst1* mutant, stems from 6-week-old plants were used. Pith, xylem and interfascicular fiber cells were laser-microdissected from non-elongating stem internodes as previously described [31] and the isolated cells were used for total RNA isolation. Real-time quantitative PCR analysis was performed using the first strand cDNA as a template. The PCR primers for *TBL32* were 5’-gaggaattagaccaaagagcagag-3’ and 5’-tcatggataaaataacttggcaaag-3’, and those for *TBL33* were 5’-tacaggaaagatgcacatacgtcg-3’ and 5’-tcaagtatagaaaagtttagcaaagag-3’. The relative expression level of each gene was calculated by normalizing their PCR threshold cycle numbers with those of three reference genes, *UBQ10*, *GAPDH*, and *EF1α*. The data were the mean of three biological replicates with three technical replicates for each sample.

### GUS Reporter Gene Analysis

The *TBL32* and *TBL33* genes containing a 3-kb 5’ upstream sequence, the entire coding region, and a 2-kb 3’ downstream sequence were ligated into the binary vector pBI101 (Clontech) to create the GUS reporter constructs. The GUS gene was placed in frame at the end of the *TBL32* and *TBL33* coding regions. Transgenic plants were generated by transforming the GUS reporter constructs into wild-type *Arabidopsis* plants using the agrobacterium-mediated transformation. GUS activity was analyzed in inflorescence stems and roots from 6-week-old plants as described previously [34]. Examination of at least 30 independent GUS plants revealed a consistent GUS staining pattern.

### Subcellular Localization

The subcellular localization of TBL32 and TBL33 was performed by co-expressing YFP-tagged TBL32 and TBL33 with CFP-tagged Golgi marker FRA8 in Arabidopsis leaf protoplasts [47]. Transfected protoplasts were observed for fluorescence signals using a confocal microscope (Leica Microsystems).

### Histology

Stems and root-hypocotyls were fixed in 1.5% formaldehyde and embedded in LR White resin (Electron Microscopy Sciences) [48]. Sections with 1-μm-thick were cut and stained with toluidine blue for light microscopy. For transmission electron microscopy, stem sections with 85-nm-thick were cut, post-stained with lead citrate and uranyl acetate, and examined under a transmission electron microscope. For each mutant, stems from at least 8 plants were sectioned and the representative data were shown. Stem breaking force was measured using a digital force tester (Larson System) [49]. The basal parts of inflorescence stems from at least 20 independent mature plants (10-week-old for wild type, *tbl32*, *tbl33*, and *tbl32 tbl33*; 12-week-old for *esk1* and *tbl32 esk1*; 14-week-old for *tbl33 esk1*; 18-week-old for *tbl32 tbl33 esk1*) were examined. The breaking force was considered as the force to break a stem segment apart.

### Cell Wall Sugar Composition Analysis

Stems from at least 50 Arabidopsis plants for each mutant line were pooled for cell wall isolation [34]. Cell wall sugars as alditol acetates were analyzed on an Agilent 6890N gas-liquid chromatography (Wilmington) equipped with a 30 m x 0.25 mm (i.d.) silica capillary column DB 225 (Alltech Assoc.) according to Hoebler et al. [50]. The Saeman hydrolysis used in this study was a preferred method for insoluble samples such as intact cell walls, whereas the TFA hydrolysis is used for soluble polysaccharides such as isolated polysaccharides, noncellulosic polysaccharides and wall fractions [51]. Furthermore, the Saeman hydrolysis and the TFA hydrolysis released the same amounts of cell wall sugars [34], and therefore, the Saeman hydrolysis is suitable for sugar composition analysis of intact cell walls used in this study. The data were the average of three separate pools of samples.

### Quantitative Measurement of Acetyl Groups

Isolation of acetylated xylan was done according to Goncalves et al. [15]. Acetic acid was released by incubating xylan with NaOH [13], and the released acetyl groups was quantitated using an acetic acid assay kit (Megazyme). The data were the average of three separate pools of samples.

### Matrix-Assisted Laser-Desorption Ionization Time-Of-Flight Mass Spectrometry (MALDI-TOF MS)

Acetylated xylan was digested by endo-1,4-β-xylanase M6 from rumen microorganism (EC 3.2.1.8; CAZY family GH10) (Megazyme) and the resulting xylooligosaccharides were examined using a MALDI-TOF mass spectrometer according to Yuan et al. [3]. Spectra were the average of 100 laser shots.

### ^1^H-NMR Spectroscopy

Acetylated xylan from various mutants was analyzed on a 600 MHz spectrometer using a 3 mm cryogenic triple resonance probe [52]. All NMR samples were prepared and run according to Yuan et al. [3] with collection of 128 transients using a spectral width of 6,000 Hz and an acquisition time of 5-seconds. The ^1^H NMR assignments of resonances were performed based on the NMR spectral data for xylan structure [12,38].

### Statistical Analysis

The data of quantitative PCR analysis and cell wall chemical analysis were subjected to statistical analysis using the Student’s *t* test program (http://www.graphpad.com/quickcalcs/ttest1.cfm), and the quantitative difference between the two groups of data for comparison in each experiment was found to be statistically significant (*p* < 0.001).

### Accession Numbers

The *Arabidopsis* Genome Initiative locus identifiers for the Arabidopsis genes investigated in this study are TBL32 (At3g11030), TBL33 (At2g40320), and ESK1 (At3g55990).
